# Starch Hydrolysis and Vessel Occlusion Related to Wilt Symptoms in Olive Stems of Susceptible Cultivars Infected by *Verticillium dahliae*

**DOI:** 10.3389/fpls.2018.00072

**Published:** 2018-01-31

**Authors:** Carlos Trapero, Esteban Alcántara, Jaime Jiménez, María C. Amaro-Ventura, Joaquín Romero, Birger Koopmann, Petr Karlovsky, Andreas von Tiedemann, Mario Pérez-Rodríguez, Francisco J. López-Escudero

**Affiliations:** ^1^Departamento de Agronomía, Universidad de Córdoba, Campus de Excelencia Internacional Agroalimentario, Córdoba, Spain; ^2^CSIRO-Agriculture and Food, Narrabri, NSW, Australia; ^3^Department of Crop Sciences, Georg-August-Universität Göttingen, Göttingen, Germany

**Keywords:** defoliating pathotype, drought stress, *Olea europaea*, *Verticillium* wilt, xylem cavitation

## Abstract

This study investigated starch content, amount of pathogen DNA and density of occluded vessels in healthy and *Verticillium dahliae* infected olive shoots and stems. Starch hydrolysis is considered a mechanism to refill xylem vessels that suffered cavitation by either, drought conditions or pathogen infections. The main objective of this work was to evaluate this mechanism in olive plants subjected to *V. dahliae* infection or to drought conditions, in order to know the importance of cavitation in the development of wilting symptoms. In initial experiments starch content in the shoots was studied in trees of cultivars differing in the level of resistance growing in fields naturally infested with *V. dahliae*. The starch content, esteemed by microscopic observation of stem transversal sections stained with lugol, decreased with the level of symptom severity. Results were confirmed in a new experiment developed with young plants of cultivars ‘Picual’ (highly susceptible), ‘Arbequina’ (moderately susceptible) and ‘Frantoio’ (resistant), growing in pots under greenhouse conditions, either inoculated or not with *V. dahliae*. In this experiment, the pathogen DNA content, quantified by real-time PCR, and the density of occluded vessels, recorded by microscopic observations of transversal sections stained with toluidine blue, were related to the symptoms severity caused by the pathogen. Finally, a drought experiment was established with young plants of the cultivar ‘Picual’ grown in pots under greenhouse conditions in order to compare the effects caused by water deficit with those caused by the pathogen infection. In both cases, results show that starch hydrolysis occurred, what indirectly evidence the importance of xylem cavitation in the development of the symptoms caused by *V. dahliae* but in the water stressed plants no vessel occlusion was detected.

## Introduction

Vascular wilt caused by the soilborne pathogen *Verticillium dahliae* Kleb., occurs in a wide range of herbaceous and woody plant species (Hiemstra and Harris, 1998; Pegg and Brady, 2002). One of the most destructive Verticillium diseases is Verticillium wilt of olive (*Olea europaea* L.) plants (Bubici and Cirulli, 2011; López-Escudero and Mercado-Blanco, 2011; Triki et al., 2011; Tsror, 2011; Mercado-Blanco and López-Escudero, 2012). This disease widely occurs in the Mediterranean Basin, particularly, in Spain where approximately 2.5 million ha of olive trees are planted (Barranco et al., 2010).

Disease control requires the application of an integrated strategy comprising, before plantation, the use of healthy plant material and non-infested soil, and after plantation, control measures aimed at reducing the present inoculums in soil, avoiding the entrance in the orchard of new infective structures, and reducing the effectiveness of this inoculum in causing disease. Among these measures, the use of resistant cultivars is likely the most important one (López-Escudero and Mercado-Blanco, 2011; Mercado-Blanco and López-Escudero, 2012; Trapero et al., 2013b). Unfortunately, almost all olive cultivars economically interesting are susceptible to the pathogen, including ‘Picual,’ the major cultivar used in Spain. Up to date, only few commercial cultivars have been identified as resistant (e.g., ‘Frantoio’), or moderately susceptible (e.g., ‘Arbequina’).

Like other vascular pathogens, *V. dahliae* colonizes xylem vessels, impairs water transport and causes wilting in some branches or throughout the shoot. Plant colonization requires conidia to ascend with the sap flow once they reach the pit cavities, and to germinate such that hyphae may transverse the pit membrane to reach the adjacent vessel (Fradin and Thomma, 2006; Báidez et al., 2007). The loss of hydraulic conductivity has been attributed to vessel occlusions induced by fungal vascular growth or plant responses (Mace et al., 1981; Beckman, 1987; Yadeta and Thomma, 2013; Gharbi et al., 2017). Tyloses and gels produced by the plant to occlude vessels are considered a part of the plant defense response halting the spread of the fungus, but if many vessels are affected, drought stress appears (Fradin and Thomma, 2006; Yadeta and Thomma, 2013).

Cavitation is another process that reduces water conductivity in the xylem due to air filling of the vessels. These vessel embolisms may be induced by water stress or frost, but they may be also caused by infections from vascular wilt pathogens (Newbanks et al., 1983; Tyree and Sperry, 1989; Pérez-Donoso et al., 2007; Martín et al., 2013; Yadeta and Thomma, 2013; Pouzoulet et al., 2014). Nevertheless, no information about cavitation induced by vascular pathogen infections in olive is available in the literature. However, in this crop, drought susceptibility has been associated with the formation of embolisms (Ennajeh et al., 2008) and the vulnerability to cavitation has been studied in relation to xylem structural characteristics (Trifiló et al., 2007).

To restore the hydraulic conductivity, an active mechanism may operate by refilling the embolized vessels with water. Degradation of starch in the parenchyma cells of the xylem produces soluble sugars that are released into the vessels, thereby promoting an osmotic flux of water into their lumen (Steudle, 2001; Brodersen and McElrone, 2013). This has been described for instances in walnut and peach trees (Améglio et al., 2002, 2004) and in Laurus (Salleo et al., 2009).

Previous microscopic observations made in our laboratory indicated that olive stems from the susceptible ‘Picual’ cultivar infected by *V. dahliae* contained less starch and more occluded vessels than those from asymptomatic stems from the same cultivar (data not published). Indeed, starch hydrolysis is considered a mechanism to refill xylem vessels that suffered cavitation by either, drought conditions or pathogen infections. The main objective of this work was to evaluate this mechanism in olive plants subjected to *V. dahliae* infection in susceptible and resistant olive cultivars or to drought conditions, in order to know the importance of cavitation in the development of wilting symptoms.

## Materials and Methods

### Experiments Conducted on Naturally Infested Fields

Experiments were carried out at fields naturally infested by *V. dahliae* located in the term of Andújar in 2012 and 2013 (Jaén province, Andalucía, southern Spain) planted with different commercial olive cultivars, such as ‘Picual,’ ‘Hojiblanca,’ ‘Cornicabra,’ ‘Frantoio,’ or ‘Arbequina.’ Sampling was made from olive trees when Verticillium wilt symptoms disease symptoms were apparent. In 2012, samples were collected from various cultivars growing in the experimental plot, whereas in 2013 they were collected from 15 year-old ‘Picual’ (susceptible cv.) trees. In 2012, three groups of samples and 10 samples per group were collected, with no consideration of the cultivar: asymptomatic shoots from asymptomatic trees, asymptomatic shoots from affected trees and dead shoots from affected trees. In 2013, the three groups of samples collected were: asymptomatic shoots (*n* = 7), affected shoots with different degrees of symptom severity (defoliation, chlorosis, and/or wilting, *n* = 21) and dead shoots (*n* = 4). For both years, samples were collected during late spring and each sample was collected from just one tree. Starch content and density of occluded vessels (only for year 2013) were determined as explained below.

### Experiments Conducted by Artificial Inoculations

Plant material used for artificial inoculations consisted of 6 month-old plants of ‘Picual,’ ‘Arbequina,’ and ‘Frantoio’ cultivars, which are considered highly susceptible, moderately susceptible and resistant, respectively, to defoliating isolates of *V. dahliae* in field conditions (Trapero et al., 2013b). In controlled conditions, both ‘Picual’ and ‘Arbequina’ are considered susceptible (López-Escudero et al., 2004). Plants were inoculated with the *V. dahliae* defoliating isolate (VCG1A) named “V117,” from the collection of the Agronomy Department, University of Córdoba (Blanco-López et al., 1984). This isolate was collected in southern Spain from a cotton plant. The high virulence in olive of this isolate has been previously reported in several artificial inoculations (López-Escudero et al., 2004, 2007; Martos-Moreno et al., 2006; Trapero et al., 2013a, 2015). Plants were inoculated by dipping their bare roots for 30 min in a 10^7^ conidia/ml suspension of the described *V. dahliae* isolate. Non-inoculated (control) plants were treated by the same way but using sterilized water. Both inoculated and control plants were then transplanted to a mix of peat, coir, and perlite (55:30:15) in sterile 1.5 L pots. Plants were grown in a greenhouse under day/night temperatures of 22 ± 3 and 18 ± 3°C, arranged in a randomized block design.

Disease severity was evaluated on a weekly basis for 10 weeks, starting 2 weeks after inoculation. Wilt resistance was assessed on a scale from 0 to 4 based on the percentage of plant tissue affected by chlorosis, leaf and shoot necrosis or defoliation (0 = healthy or asymptomatic plant; 1 = plant affected by 1–33%; 2 = 34–66%; 3 = 67–99%; 4 = dead plant) (López-Escudero et al., 2004). For each cultivar, 41 plants were inoculated and 41 treated as a control (non-inoculated). To record the disease severity, 14 of the inoculated plants and 14 of the non-inoculated plants per cultivar were kept until the end of the experiment, evaluating weekly the disease severity from the 4th to the 15th weeks after inoculation. To determine the pathogen DNA amount, starch content and density of occluded vessels, the remaining 27 inoculated plants and 27 non-inoculated plants per cultivar were used. For the pathogen DNA, three inoculated and three control plants per cultivar were collected and sampled at different times: just before inoculation and at 1, 3, 5, 7, 9, 11, 13, and 15 weeks after inoculation. For both starch content and density of occluded vessels the same plants were used, but they were grouped by symptom categories. The determinations were only made from the time at which disease symptoms started, that is at 7, 9, 11, 13, and 15 weeks after inoculation.

### Experiment on Plants Subjected to Drought Stress

In this experiment, only ‘Picual’ plants were used, which were grown under the same greenhouse conditions described before, but without inoculating plants. Instead, plants were subjected to three different irrigation treatments.

One group of five plants was watered daily (control plants) and a second group of six plants remained without irrigation during 10 days and showed wilting symptoms (drought stressed plants), at this time starch content and density of occluded vessels were determined in both groups. A third group of six plants remained without irrigation during 10 days and after that they received daily irrigation during a period of 14 days (post-drought-irrigated plants), at this time starch content and density of occluded vessels were determined as explained below.

### Determination of Starch Content, Density of Occluded Vessels, and Pathogen DNA

For assessing the starch content, from each sampled plant we cut a 2 cm long portion from the middle of the stem, then with a hand microtome three transversal sections, approximately 30 μm thick, were obtained and subsequently stained with Lugol (2 g KI and 1 g I_2_ dissolved in 100 ml distilled water) for 3 min. The slides were observed using a Nikon YS100 light microscope and the starch content in the different tissues (pitch, xylem, phloem, and cortex) were assessed on the basis of a 0 to 3 rating scale: 0 = less than 10%; 1 = from 10 to 30%; 2 = from 30 to 60%; 3 = from 60 to 100% stained tissue. To obtain a more objective estimation, the samples were blind-analyzed.

The density of occluded vessels in the xylem of the stem was determined by light microscopy observation following a similar procedure than that for starch content. In this case, sections were stained with toluidine blue at 0.05% (Sakai, 1973) for 3 min. The number of occluded vessels was counted using a 40x magnification objective and referred to the xylem area (n°/mm^2^). This area was calculated from data of two perpendicular diameters of both the internal and external limits of the xylem. Partially occluded vessels were observed, but only the completely occluded vessels were counted.

For quantifying the amount of pathogen DNA in the experiment of artificial inoculation, one sample was taken per plant. Each sample of 0.5 g shoot tissues was composed by 10 1 cm-long shoot segments randomly taken from a single plant. Samples were immersed in liquid nitrogen for 10 min. Subsequently, the frozen samples were lyophilized for 24 h, ground into a fine powder using a freezer mill (Spex 6770, SPEX SamplePrep, Metuchen, NJ, United States), and stored at -80°C prior to use. The pathogen DNA was quantified using real-time PCR. First, DNA was extracted from the samples using the DNeasy Plant Mini Kit from Qiagen (Hilden, Germany). Primers sequences and amplification conditions were described by Eynck et al. (2007), reaction buffer was modified by adding 0.3 μM bovine serum albumin (Sigma-Aldrich, St. Louis, MO, United States) to the reaction mixture which was 10 μl in volume. Real-time PCR amplification and melting curve analysis were performed using the iCycler System (Bio-Rad, Hercules, CA, United States). The results were analyzed with the Bio-Rad iQ5 program (Bio-Rad, Hercules, CA, United States). The amount of *V. dahliae* DNA was estimated from a calibration curve using increasing amounts of genomic *V. dahliae* DNA from 0.5 to 64.0 pg. The concentration of *V. dahliae* DNA used for the construction of the calibration curve was estimated using densitometry on agarose gels stained with ethidium bromide, with Lambda Phage DNA as the standard.

### Statistical Analyses

The starch content and density of the occluded vessels were analyzed using an analysis of variance (ANOVA) performed by Statistix 9.0 (Analytical Software, Tallahassee, FL, United States). The mean values were compared using the Fisher’s protected LSD test at *P* = 0.05. Due to a lack of homogeneity in the variances, the values for disease severity were analyzed using a non-parametric Kruskal–Wallis test at *P* = 0.05.

## Results

### Starch Content in Experiments Conducted on Naturally Infested Fields

**FIGURE 1:**
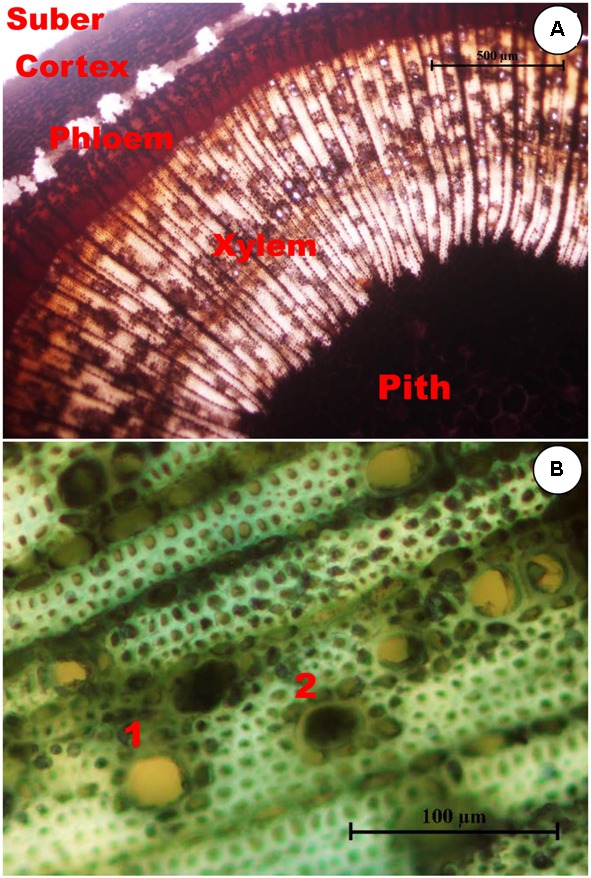
**(A)** Transverse section of an olive stem stained with Lugol showing starch accumulation. Cells with starch accumulation, stained black inside, are abundant in the pith, axial, and radial systems of both the xylem and phloem, and in the cortex, but they are absent in the suber. **(B)** Xylem in the transverse section of an olive stem stained with toluidine blue showing occlusions in the vessels. Non-occluded vessels, such as 1, can be distinguished from occluded vessels, such as 2.

**FIGURE 2:**
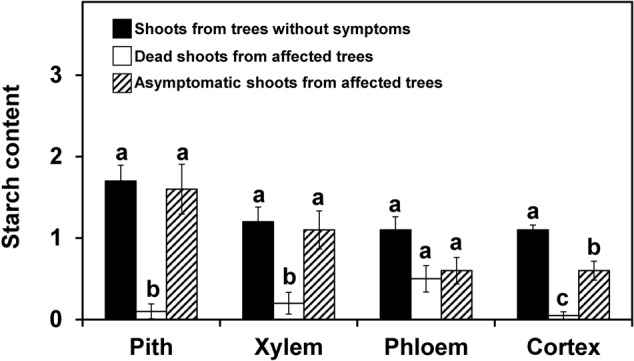
Starch content in different stem tissues of olive shoots (2012 experiment on naturally infested fields). The shoots were collected from trees without symptoms and from asymptomatic or dead zones of trees affected by *Verticillium dahliae* during field surveys in 2012, and starch content was assessed according to a 0-to-3 scale using a microscope. The values represent the mean of 10 shoots and columns with different letters in each tissue are significantly different according to Fisher’s protected LSD test at *P* = 0.05. Bars represent the mean (±) standard errors.

**FIGURE 3:**
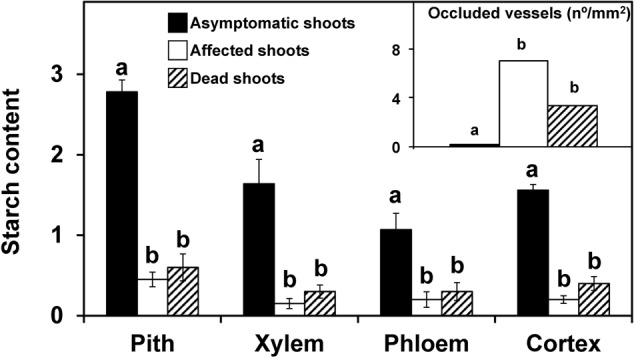
Starch content in different stem tissues and the density of occluded vessels (inside figure) of olive shoots (2013 experiment). Asymptomatic (*n* = 7), affected (*n* = 21), and dead (*n* = 4) shoots were collected in an olive orchard of the ‘Picual’ cultivar affected by *V. dahliae* during field surveys conducted in 2013. Starch content was assessed according to a 0-to-3 scale using a microscope. Density of occluded vessels was quantified using a microscope. Columns with different letters in each tissue were significantly different according to Fisher’s protected LSD test at *P* = 0.05. Bars represent the mean (±) standard errors.

Shoots showing no symptoms, from both asymtomatic or symptomatic trees, contained abundant starch in the stem, which was distributed in the pith, xylem (radial and axial parenchyma), phloem (radial and axial parenchyma) and cortex (**Figure 1A**). For both years and for most of the tissues studied, the starch content in the symptomatic shoots was significantly lower (*P* < 0.05) than in the asymptomatic shoots (**Figures 2, 3**). In the 2012 sampling, no differences were observed between the two groups of non-symptomatic samples with the exception of the cortex (**Figure 2**). In the 2013 sampling, the symptomatic and dead shoots had significantly lower starch content in all stem tissues compared with asymptomatic shoots (**Figure 3**). In the same way, the density of occluded vessels in the affected or dead shoots was significantly (*P* < 0.05) higher than that assessed in the asymptomatic shoots (**Figure 3**, inside). **Figure 1B** illustrates the differences between the occluded and non-occluded vessels in the microscopic observations.

### Starch Content, Density of Occluded Vessels, Disease Symptoms, and Pathogen DNA in Experiments Conducted by Artificial Inoculations

**FIGURE 4:**
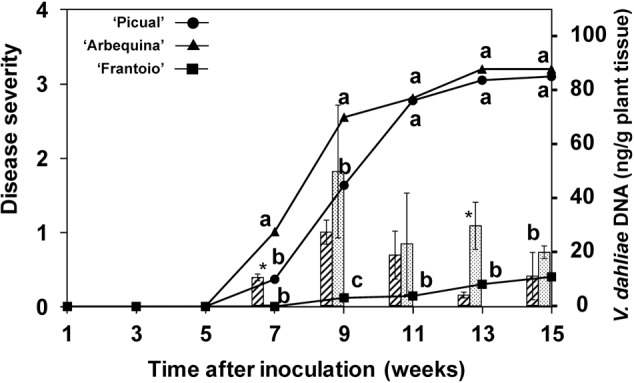
Progress of disease severity and amount of *V. dahliae* DNA in plants of ‘Picual,’ ‘Arbequina,’ and ‘Frantoio’ cultivars inoculated with *V. dahliae* (experiment of artificial inoculation). The disease severity values are the means of 14 replicates in each week according to a 0-to-4 scale. Columns represent the average amount of *V. dahliae* DNA in three sampled plants (hatched columns for “Arbequina’ and pointed columns for ‘Picual’). Different letters indicate significant differences in disease severity according to a non-parametric Kruskal–Wallis test at *P* = 0.05. An asterisk indicates significant difference in the amount of DNA between ‘Arbequina’ and ‘Picual’ according to according to Fisher’s protected LSD test at *P* = 0.05. Bars represent the mean (±) standard errors.

The disease progression is shown in **Figure 4**. The susceptible ‘Arbequina’ and ‘Picual’ cultivars’ showed symptoms since the 5th week after inoculation and rapidly developed symptoms between the 6th and the 11th weeks, reaching a final severity around 3. In contrast, symptoms of the resistant ‘Frantoio’ cultivar started after the 9th week following inoculation and reached a final severity below 0.5. The percentage of plants with symptoms was 100% in ‘Picual,’ 76% in ‘Arbequina,’ and 11% in ‘Frantoio.’

Pathogen DNA in plant tissue was quantified by real-time PCR. Inhibition of PCR by plant matrix was excluded by comparing threshold cycle values for pure fungal DNA with fungal DNA mixed with control plant DNA (data not shown). *V. dahliae* DNA was only detected in inoculated plants, with DNA amounts that were significantly higher in the susceptible cultivars from the 7th week after inoculation (*P* = 0.0039). At this point in time, the amount of pathogen DNA in the stems of the ‘Arbequina’ and ‘Picual’ inoculated plants was 0.3 and 10.4 ng/g of plant tissue, respectively, and increased until the 9th week after inoculation in concurrence with a period of rapid symptom development (**Figure 4**). Afterward, the DNA amount slowly decreased to 10.9 and 20.7 ng/g of plant tissue in ‘Arbequina’ and ‘Picual,’ reaching low values in dead plants (plants with disease severity value = 4). In cultivar ‘Frantoio,’ fungal DNA was only detected in one of all the sampled inoculated plants.

**FIGURE 5:**
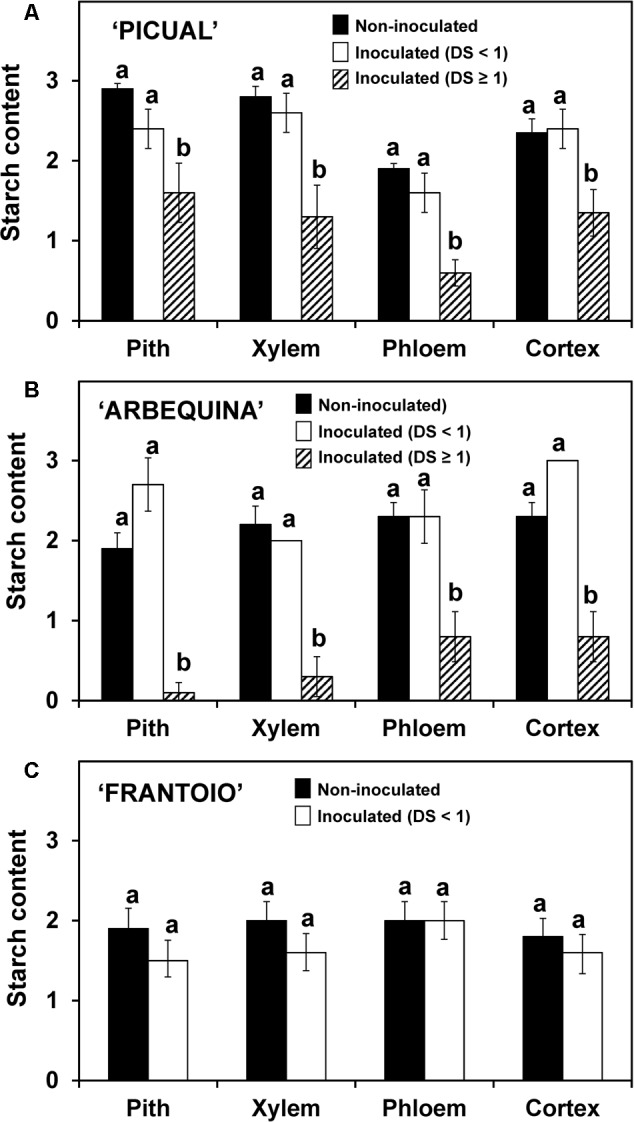
Starch content in different stem tissues of ‘Picual’ **(A)**, ‘Arbequina’ **(B)**, and ‘Frantoio’ **(C)** cultivars not inoculated or inoculated with *V. dahliae* (experiment of artificial inoculation). Three groups of plants were compared: non-inoculated (*n* = 15), inoculated with Disease Severity (DS) < 1 (*n* = 4 for ‘Picual,’ *n* = 5 for ‘Arbequina,’ and *n* = 15 for ‘Frantoio’) and inoculated with Disease Severity (DS) ≥ 1 (*n* = 11 for ‘Picual,’ *n* = 10 for ‘Arbequina,’ and *n* = 0 for ‘Frantoio’). Starch content was assessed according to a 0-to-3 scale using a microscope. Columns with different letters in each tissue are significantly different according to Fisher’s protected LSD test at *P* = 0.05. Bars represent the mean (±) standard errors.

The starch content and the density of occluded vessels were determined in the same plants used for pathogen DNA, but in this case results are presented according to the severity reached by each plant, independently of the time after the inoculation. In the three cultivars, the starch content in the inoculated plants that showed slight disease symptoms (lower than 1 in the rating scale) did not significantly differ from the non-inoculated plants (**Figure 5**). On the other hand, in those plants with more severe symptoms (severity rating ≥ 1, only found in ‘Picual’ and ‘Arbequina’ cultivars), a significant reduction in starch content was observed.

**FIGURE 6:**
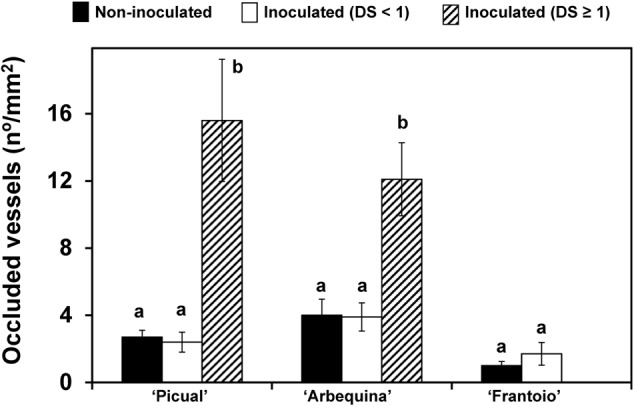
Density of occluded vessels in plants of ‘Picual,’ ‘Arbequina,’ and ‘Frantoio’ cultivars non-inoculated or inoculated with *V. dahliae* (experiment of artificial inoculation). Plants are grouped according to the level of disease severity shown: non-inoculated, inoculated with Disease Severity (DS) < 1 and inoculated with Disease Severity (DS) ≥ 1. Density of occluded vessels was quantified using a microscope. Columns with different letters for each cultivar are significantly different according to Fisher’s protected LSD test at *P* = 0.05. Bars represent the mean (±) standard errors.

Density of occluded vessels was also related to disease severity (**Figure 6**). Non-inoculated plants and those inoculated but asymptomatic (disease severity = 0) showed a significantly (*P* < 0.05) lower density of occluded vessels than inoculated plants that were more severely affected (disease severity ≥ 1). These plants, from the ‘Picual’ and ‘Arbequina’ cultivars, reached mean values higher than 12 occluded vessels per mm^2^ of xylem, whereas in the resistant ‘Frantoio’ cultivar, the mean values were always below 2.

### Starch Content and Occluded Vessels in the Drought Stress Experiment

**FIGURE 7:**
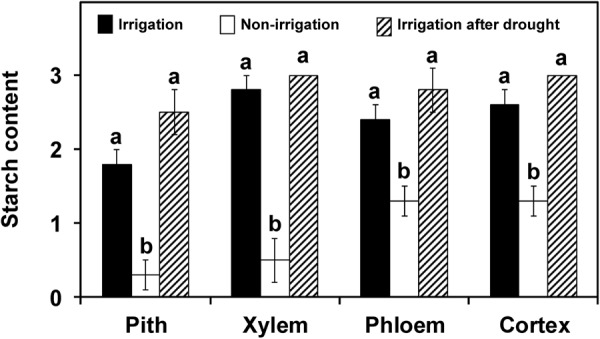
Starch content in the different stem tissues of plants of ‘Picual’ cultivar subjected to three different irrigation treatments (drought stress experiment). Values are the mean of 5, 6, and 4 plants from the irrigation treatments: non-irrigation and irrigation after drought, respectively. Columns with different letters in each tissue are significantly different according to the Fisher’s protected LSD test at *P* = 0.05. Xylem vessel occlusions were not detected in any of the groups.

Plants exposed to 10 days without watering showed wilt symptoms and a significantly (*P* < 0.05) lower starch content in their stems than plants that had received daily irrigation (**Figure 7**). This decrease was exhibited in all tissues, and especially in the pith and xylem. In plants exposed to 10 days without watering followed by daily irrigation over a period of 14 days, recovery from wilting symptoms was observed in four out of six plants. The starch content in the plants showing recovery was similar to that in plants with continuous adequate irrigation (**Figure 7**), whereas in plants that did not show recovery, the starch content was similar to that found in plants that underwent 10 days without irrigation (data no shown). On the other hand, xylem vessel occlusions were not detected in any of the three groups of plants.

## Discussion

Both experiments conducted on naturally infested soils or in artificial inoculations performed in this work showed that the starch content in the stems of olive plants decreased when they are infected and express wilting symptoms caused by *Verticillium* infection. A similar effect was found when olive plants were subjected to drought stress, although after being watered again they were able to recover from wilt symptoms and reestablish a similar starch content to that of unstressed plants.

A decrease in the starch content has been previously correlated with vessel cavitation as an active response to restoring xylem function by refilling the vessels with water through osmotic gradients generated by the production of soluble sugars (Steudle, 2001; Améglio et al., 2002, 2004; Salleo et al., 2009). This starch response has been shown for embolisms induced by frost (Améglio et al., 2002, 2004), by water stress (Salleo et al., 2009), and by pathogen infections, as also indicated in this study.

Starch reserves may be used for protecting the plant against unfavorable conditions, such as drought (O’Brien et al., 2014) or frost (Essiamah and Eschrich, 1985). In olives, Bustan et al. (2011) concluded that carbohydrate reserves may play a significant role in promoting survival under the naturally unpredictable Mediterranean climate, with one of the main function of soluble sugars being the refilling of cavitated vessels. The lower starch content found in olive plants subjected to drought stress compared to well-watered plants, as well as its recovery after resumption of irrigation, supports this role of starch. We also found that starch hydrolysis was not only localized in the parenchyma cells of the xylem but also in the pith, the phloem, and the cortex; similarly to what happens in several tree species during dormancy (Essiamah and Eschrich, 1985).

The low starch content found in olive plants with severe Verticillium wilt symptoms under both field and greenhouse conditions strongly suggests that embolisms induced by the pathogen play a critical role in the development of symptoms, as has been proposed for other vascular wilt pathogens (Yadeta and Thomma, 2013; Pouzoulet et al., 2014). An example is the important proportion of vessels that become progressively embolized in grapevine shoots after infection by the bacterium *Xylella fastidiosa* (Pérez-Donoso et al., 2007). Newbanks et al. (1983) also detected embolized vessels in American elms infected by *Ceratocystis ulmi* and considered them to be the primary cause for the loss of hydraulic conductivity, whereas vessel occlusions are considered to be a secondary phenomenon. Pathogens could increase the risk of cavitation by several processes, such as a decrease in the surface tension of the xylem sap caused by pathogen produced compounds (Tyree and Sperry, 1989; Solla and Gil, 2002) or by altering the structure of the pit membrane when trying to colonize adjacent vessels (Tyree and Sperry, 1989; Steudle, 2001; Pérez-Donoso et al., 2007; Brodersen et al., 2010, 2014; Pouzoulet et al., 2014). In order to spread, conidia from *Verticillium* spp. trapped in the pit chamber germinate and the hyphae transverse the pit membrane by degrading its structure with pectinolytic enzymes (Fradin and Thomma, 2006; Báidez et al., 2007), thereby increasing the risk of cavitation.

Besides the reduced starch content, we also showed that Verticillium wilted shoots exhibited a high density of occluded vessels on susceptible olive cultivars. The vessels occluded by gels and tyloses have been previously described in stems of ‘Picual’ infected by *V. dahliae* (Báidez et al., 2007). Though vessel occlusion can be considered a mechanism to limit pathogen spread, the high density found in the susceptible cultivars suggests that they are just a consequence of cavitation, as suggested by others (Newbanks et al., 1983; Tyree and Sperry, 1989; Pérez-Donoso et al., 2007; Brodersen et al., 2010; Martín et al., 2013). In the resistant ‘Frantoio’ cultivar, the infected plants had limited symptoms and the starch content and density of occluded vessels in the stem did not differ from the control plants. This may be explained by the quick defense response and activation of physical and chemical mechanisms in the root and basal stem (Fradin and Thomma, 2006; Bubici and Cirulli, 2012; Cabanás et al., 2015) that restricted the shoot colonization and, consequently, the effect on vessel cavitation and occlusion. On the other hand, no vessel occlusion was detected in drought-stressed plants. In American elm infected with *C. ulmi* hydraulic conductivity was disrupted without visible colonization and/or occlusion of xylem vessels (Newbanks et al., 1983). These results may be explained because vessel occlusion after cavitation take a time that was not achieved, at least in our drought treatment. The disease severity is assumed to reflect the intensity of colonization by the pathogen. *V. dahliae* seems to be able to intensively colonize the stems of ‘Arbequina’ and ‘Picual,’ at the same time that causes acute symptoms on them. Although ‘Arbequina’ is reported to be slightly more resistant than ‘Picual’ in field conditions, both cultivars are reported to be very susceptible under controlled conditions (López-Escudero et al., 2004) and they performed this way in the present study. On the other hand, in cultivar ‘Frantoio’ that is reported to be resistant in both controlled (López-Escudero et al., 2004) and field conditions (Trapero et al., 2013b), symptoms were minimal and the DNA was detected only at very low levels. Similar differences in DNA among susceptible and resistant olive cultivars have been previously reported (Mercado-Blanco et al., 2003). In inoculated plants, variability in the amount of *V. dahliae* DNA between samples and within the same plant has been described (Mercado-Blanco et al., 2003; Markakis et al., 2009). In the present work, considerable variability in *V. dahliae* DNA content between plants was also observed, however, in general, an increase in the amount of DNA was found during a period of rapid development of symptoms followed by a period during which it progressively decreased, especially in dead shoots. This decrease of pathogen biomass was also observed in previous studies (Mercado-Blanco et al., 2003; Markakis et al., 2009; Gharbi et al., 2016) in both resistant and susceptible olive cultivars once the disease is fully developed. This may be due to partial lysis of the hyphae and propagules of the fungus, as suggested by Mercado-Blanco et al. (2003) and Gramaje et al. (2013).

## Conclusion

We hypothesize that cavitation plays a key role determining the development of symptoms caused by *V. dahliae.* In susceptible cultivars, widespread colonization of the xylem by the pathogen apparently causes extensive cavitation that, in turn, may instigate the hydrolysis of starch to refill vessels. On the other hand, in resistant cultivars, the pathogen is not able to extensively colonize the xylem and therefore cavitation and starch hydrolysis would not occur to a great extent.

## Author Contributions

Conceived and designed the experiments: CT, EA, and FL-E. Performed the field experiments: CT, EA, FL-E, JJ, and MP-R. Performed the glasshouse experiments and laboratory analysis: CT, JJ, MA-V, MP-R, and JR. Analyzed the data: CT, EA, JJ, and FL-E. Contributed reagents/materials/analysis tools: AvT, BK, EA, and FL-E. Drafted the manuscript: CT, EA, and FL-E. Edited the manuscript and contributed to data analysis: AT, BK, JR, and PK. All authors read and approved the manuscript.

## Conflict of Interest Statement

The authors declare that the research was conducted in the absence of any commercial or financial relationships that could be construed as a potential conflict of interest.
